# Gut Microbiota, Intestinal Barrier Function, and Metabolism Across Adiposity and Glucose Tolerance

**DOI:** 10.3390/nu17213380

**Published:** 2025-10-28

**Authors:** Karynne Grutter Lopes, Maria das Graças Coelho de Souza, Fernanda de Azevedo Marques Lopes, Vicente Lopes da Silva Júnior, Ana Teresa Pugas Carvalho, Davy Carlos Mendes Rapozo, Carolina Monteiro de Lemos Barbosa, Eliete Bouskela, Raquel Carvalho Castiglione, Rodolpho Matos Albano, Luiz Guilherme Kraemer-Aguiar

**Affiliations:** 1 Postgraduate Program in Clinical and Experimental Physiopathology (Fisclinex), Faculty of Medical Sciences, State University of Rio de Janeiro, Rio de Janeiro 20550-013, Brazil; kjgolrj@gmail.com (K.G.L.); mgcsouza@gmail.com (M.d.G.C.d.S.); fernandamar@gmail.com (F.d.A.M.L.); vlsjunior@uol.com.br (V.L.d.S.J.); eliete.bouskela@gmail.com (E.B.); 2 Laboratory for Clinical and Experimental Research on Vascular Biology (Biovasc), Biomedical Center, State University of Rio de Janeiro, Rio de Janeiro 20550-013, Brazil; carolmlbarbosa@gmail.com (C.M.d.L.B.); rccastiglione@gmail.com (R.C.C.); 3 Department of Physiological Sciences, Roberto Alcantara Gomes Biology Institute, State University of Rio de Janeiro, Rio de Janeiro 20550-013, Brazil; 4 Multiuser Clinical Research Center (CePeM), Pedro Ernesto University Hospital (HUPE), State University of Rio de Janeiro, Rio de Janeiro 20550-013, Brazil; atpugas@yahoo.com.br; 5 Gastroenterology, Department of Internal Medicine, Faculty of Medical Sciences, State University of Rio de Janeiro, Rio de Janeiro 20550-013, Brazil; 6 Research Center, Brazilian National Cancer Institute (INCA), Rio de Janeiro 20231-050, Brazil; davyrapozo@gmail.com; 7 Department of Biochemistry, Roberto Alcântara Gomes Biological Institute, State University of Rio de Janeiro, Rio de Janeiro 20550-013, Brazil; rodolphalb@gmail.com; 8 Endocrinology, Department of Internal Medicine, Faculty of Medical Sciences, State University of Rio de Janeiro, Rio de Janeiro 20550-013, Brazil

**Keywords:** obesity, inflammation, intestinal epithelial permeability, microbiota, β-actin

## Abstract

**Background/Objectives:** Obesity and dysglycemia are increasingly associated with intestinal barrier dysfunction and alterations in gut microbiota. Intestinal hyperpermeability is emerging as a therapeutic target in metabolic disorders, but human data integrating barrier biomarkers, epithelial morphology, and microbial composition remain scarce. **Methods:** Forty-six adults (82.6% female; 38.3 ± 7.8 years) were stratified into lean normoglycemic controls (CON), individuals with obesity and normoglycemia (NOB), and those with obesity and dysglycemia (DOB). Biochemical/inflammatory biomarkers, such as lipopolysaccharide (LPS) and LPS-binding protein (LBP), were measured. Duodenal biopsies were obtained by upper digestive videoendoscopy. Histomorphometry, expression of junctional and cytoskeletal proteins, and enzymatic activity of the duodenal epithelium were used as markers of intestinal permeability. Fecal microbiota composition (FMC) was analyzed by amplifying the V4 region of the 16S rRNA gene, which was sequenced using next-generation sequencing technology. **Results:** Duodenal histomorphometry did not differ across groups. Intestinal alkaline phosphatase (IAP) was significantly lower in DOB compared to CON. LPS correlated positively with fat mass, and LBP with the waist-to-hip ratio. The villus-to-crypt ratio correlated negatively with BMI, while IAP correlated inversely with fasting glucose and HbA1c. β-actin expression was inversely associated with BMI, glucose, insulin, and HOMA-IR. Microbiota diversity indices were similar between groups, although specific taxa, particularly within the Clostridiales order, were reduced in dysglycemia. **Conclusions:** Reduced IAP activity and consistent correlations between barrier biomarkers and metabolic parameters highlight intestinal barrier dysfunction as a relevant feature of obesity and dysglycemia. Subtle microbiota alterations further support a link between gut ecology and metabolic control. These findings underscore the intestinal barrier as a promising therapeutic target in metabolic disorders.

## 1. Introduction

Obesity is a major global public health challenge in the 21st century [1], contributing to an increased risk of cardiovascular diseases (CVD), type 2 diabetes *mellitus* (T2D), cancer, and premature mortality. Its development is complex and multifactorial, driven by interactions between genetic, metabolic, and environmental factors [2]. Emerging evidence suggests that the human gut microbiota plays a crucial role in obesity, metabolic syndrome, and a low-grade inflammatory status [3].

Individuals with obesity and healthy controls unsurprisingly differ in anthropometric and biochemical aspects, but they also exhibit distinct intestinal microbiota compositions [3], which partially mediate the pathogenesis of obesity [4]. Research suggests that gut microbiota influences energy extraction from food, gene expression, metabolic homeostasis, the brain–gut axis, and the endocannabinoid system [5]. Moreover, in obesity, gut microbiota alterations increase intestinal permeability and circulating endotoxins, contributing to systemic low-grade inflammation and insulin resistance [6].

Altered intestinal permeability has been observed in individuals with obesity and dysglycemia [7]; however, the mechanisms by which gut microbiota disrupts the epithelial barrier have not yet been fully clarified. In obesity, there is a relative increase in the Firmicutes phylum, comprising Gram-positive bacteria, which paradoxically leads to elevated circulating lipopolysaccharide (LPS) levels derived from the outer membranes of Gram-negative bacteria [8]. Higher LPS levels impair the intestinal epithelial barrier by promoting inflammatory cytokine release and downregulating tight junction proteins [9]. This increased LPS translocation exacerbates epithelial barrier dysfunction, allowing pathogens and toxins to enter the bloodstream, further fueling subclinical inflammation and metabolic disturbances [10,11]. Another mechanism associated with increased intestinal permeability is the reduced production of butyrate resulting from dysbiosis. Butyrate, a short-chain fatty acid synthesized by commensal bacteria [12], plays a key role in regulating the intestinal immune response by inhibiting the expression of pro-inflammatory cytokines and the activation of several pro-inflammatory signaling pathways [13]. This action mitigates intestinal inflammation and prevents its propagation into the systemic circulation [12]. In addition, butyrate contributes to the maintenance of tight junction integrity, thereby preserving gut barrier function [12]. Conversely, butyrate deficiency diminishes the intestine’s anti-inflammatory capacity and disrupts gut barrier function, resulting in increased intestinal permeability and the perpetuation of a systemic low-grade inflammatory state. Intestinal alkaline phosphatase (IAP) is an intestinal epithelial enzyme involved in maintaining gut barrier integrity, detoxifying lipopolysaccharides, and modulating inflammation, making it directly relevant to intestinal permeability and metabolic regulation. IAP plays a crucial role in gut homeostasis by inactivating LPS, thereby preventing its translocation into the bloodstream [14]. As a key factor in maintaining intestinal epithelial integrity, IAP activity is reduced in animal models of obesity and diabetes, yet it still appears to sustain bacterial diversity in the colon [15]. Notably, studies on IAP-deficient mice have shown increased LPS levels and intestinal permeability. Remarkably, supplementing their diet with IAP reversed the metabolic syndrome phenotype, highlighting its potential therapeutic role [16]. β-actin has been increasingly recognized as a structural marker of cytoskeletal integrity, and its detection in fecal samples has been proposed as a non-invasive indicator of epithelial cell shedding and barrier dysfunction. Therefore, IAP and β-actin provide complementary information on intestinal barrier status, which is central to this investigation.

Despite growing evidence linking gut microbiota and intestinal permeability to obesity, it remains unclear whether these changes are a cause or a consequence of metabolic dysfunction [5]. In particular, there is limited evidence integrating intestinal permeability biomarkers such as IAP and β-actin with both microbial and metabolic profiles across the spectrum from normoglycemia to dysglycemia. Moreover, their impact on the inflammatory profile associated with increased adiposity is not fully understood. We hypothesize that greater glucose intolerance and obesity are associated with higher epithelial barrier dysfunction and changes in fecal microbiota composition (FMC). Therefore, we investigated the metabolic and inflammatory biomarkers, intestinal permeability, and FMC in normoglycemic controls versus normoglycemic and dysglycemic individuals with obesity. Specifically, we examined the associations between intestinal permeability and metabolic markers. Our study provides a multidimensional analysis linking epithelial barrier status, gut microbial composition, and metabolic/inflammatory traits, thereby offering new insights into gut–metabolism interactions.

## 2. Materials and Methods

### 2.1. Subjects

Forty-six individuals were recruited at the outpatient care unit [82.6% female; aged 38.3 ± 7.8 years; BMI 32.6 ± 5.1 kg/m^2^] and were allocated into three groups according to body adiposity and glucose tolerance: healthy or overweight controls with normoglycemia (CON, n = 16), subjects with moderate-to-severe obesity and normoglycemia (NOB, n = 15) or those with obesity and dysglycemia (DOB, n = 15).

For all individuals, the inclusion criteria were age between 18 and 50 years, and classification based on BMI and the oral glucose tolerance test (OGTT) which divided patients into three groups: 1. CON—BMI between 20 and 27.5 kg/m^2^ and normoglycemia; 2. NOB—BMI between 30 and 40 kg/m^2^ and normoglycemia and 3. DOB—BMI between 30 and 40 kg/m^2^ and dysglycemia. Participants were classified according to both BMI and glucose tolerance status to enable comparisons between individuals with obesity with and without dysglycemia, and to isolate the specific contribution of glucose metabolism from that of adiposity. Participants in the dysglycemic group were newly diagnosed and not under pharmacological treatment for glucose control at the time of sample collection. Exclusion criteria included chronic obstructive pulmonary, kidney, liver, hematologic, or gastrointestinal diseases, uncontrolled hypertension, unstable dietary history (defined as major dietary changes during the previous month, as assessed by a trained nutritionist), and the use of probiotics, antibiotics, oral corticosteroids, cytokines, methotrexate or cytotoxic immunosuppressive agents in the past six months. Additional exclusion criteria were pregnancy or lactation, smoking, alcoholism, and infection with HIV and hepatitis B or C.

Subject recruitment, pre-participation screening, and data collection were conducted over one year. According to the OGTT, glucose tolerance was classified as follows: 1. Normoglycemia—fasting plasma glucose (PG) < 100 mg/dL or 2 h PG after 75 g oral glucose anhydrous < 140 mg/dL; 2. Dysglycemia—fasting PG between 100 and 125 mg/dL or 2 h PG > 140 mg/dL [17]. A glycated hemoglobin (HbA1c) level > 5.7% was also used as a criterion for defining dysglycemia.

### 2.2. Ethical Approval and Experimental Design

This cross-sectional study was conducted at the Laboratory for Clinical and Experimental Research on Vascular Biology (BIOVASC) and Pedro Ernesto University Hospital (HUPE), both located at the State University of Rio de Janeiro (UERJ), Rio de Janeiro, Brazil. Our study protocol was approved by the local ethics committee (CAAE: 22032113.9.0000.5259) and was registered on ClinicalTrials.gov (NCT03178006). A detailed explanation of the study was given to all participants before they provided written informed consent. All procedures were performed according to the principles outlined in the Declaration of Helsinki.

All study visits took place over three non-consecutive days, with at least 24 to 48 h between each visit, in the following order: (a) Day 1—clinical history, physical examination, anthropometric measurements, and dietary assessment; (b) Day 2—body composition assessment and collection of fecal and blood samples; (c) Day 3—upper digestive videoendoscopy (UDV) with duodenal biopsy.

### 2.3. Anthropometry, Body Composition, and Dietary Profile

Body mass and height were assessed using an electronic scale and stadiometer (Filizola^®^, São Paulo, Brazil). BMI was calculated accordingly. Waist and hip circumferences were measured with a flexible tape at the end of a normal expiration, at the midpoint between the last costal arch and the iliac crest, and at the point of the greatest circumference of the gluteal region, respectively. The waist-to-hip ratio was calculated. Total lean and fat mass percentages were determined using a bioimpedance analyzer (Biodynamics 450 Biodynamics Corporation, Shoreline, WA, USA). Dietary intake was assessed using a validated food frequency questionnaire [18]. The questionnaire covered major food groups and estimated daily energy and macronutrient intake, characterizing overall dietary habits without stratifying participants into subgroups.

### 2.4. Analyses of Metabolic and Intestinal Permeability Biomarkers

Blood samples were collected by venipuncture into serum, plasma EDTA, and plasma-fluoride tubes. Plasma-EDTA tubes were centrifuged at 1000× *g* at 4 °C for 10 min, while the plasma-fluoride and serum were centrifuged at 3000× *g* rpm at 18 °C for 10 min. Plasma and serum samples were transferred into cryotubes and stored at −80 °C until analysis. Serum LPS and LPS-binding protein (LBP) concentrations were determined using the Chromogenic End-Point Assay—QCL1000 Limulus Amebocyte Lysate (Lonza, Walkersville, MD, USA) and the human LBP ELISA kit (Hycult Biotech, Uden, The Netherlands), respectively. The sensitivity for LPS analysis was 0.10 EU/mL, and intra- and interassay coefficients of variation were <7.61% and 10.56%, respectively. For the LBP assay, the sensitivity was 4.4 μg/mL and within- and between-assay precisions were <3.12% and 8.17%, respectively. Insulin was evaluated by the Milliplex^®^ MAP Human Metabolic Hormone Magnetic Bead Panel (Merck-Millipore, Billerica, MA, USA). The sensitivity of this assay was 18.57 pg/mL, and intra- and inter-assay coefficients of variation were <10% and 20%, respectively.

Glycated hemoglobin type A1c (HbA1c) and plasma glucose levels were measured using turbidimetric inhibition immunoassay and glucose oxidase colorimetric methods, respectively. Triglycerides, total cholesterol, and high-density lipoprotein cholesterol (HDL-cholesterol) were determined using glycerol phosphate oxidase/peroxidase, cholesterol oxidase/peroxidase, and direct colorimetric methods, respectively. All biochemical analyses were performed using an Automatic Analyzer A25 (BioSystems, Barcelona, Spain). Low-density lipoprotein cholesterol (LDL-cholesterol) was calculated using the Friedewald equation [19]. The homeostatic model assessment for insulin resistance (HOMA-IR) was calculated to quantify fasting insulin resistance [(PG in mmol/L × insulin in μUI/mL)/22.5] [20]. Alanine aminotransferase (ALT) and aspartate aminotransferase (AST) levels were determined according to the International Federation of Clinical Chemistry (IFCC) method and the IFCC method without pyridoxal phosphate, respectively. The intra and inter-assay coefficients of variation for biochemical analyses were <15%. All analyses were performed according to the manufacturers’ instructions for each kit.

### 2.5. Histomorphometry, Protein Expression, and Enzymatic Activity of Intestinal Epithelium

On day 3, after a 12 h fast, participants underwent an UDV (Fujinon EG-410HR, Fuji Photo Optica, Uetake, Omiya, Japan) for tissue collection. Ten biopsy samples were obtained from the second portion of the duodenum using jumbo calipers. Part of each sample was preserved in 10% formalin and embedded in paraffin. The sections were stained with hematoxylin and eosin, and the slides were photographed using an optical microscope equipped with a digital camera (100× magnification). The obtained images were transferred to a computer, processed, and analyzed with ImageJ software version 1.54 p (National Institutes of Health, Redmond, Washington, DC, USA). Histomorphometric analysis included the following parameters: total epithelial thickness, intestinal villus height, villus diameter I, villus diameter II, crypt height, and villus-to-crypt ratio. The remaining samples were placed in cryotubes and immediately immersed in liquid nitrogen for subsequent quantification of protein expression and enzymatic activity.

A total of 50 μg of protein was subjected to electrophoresis on10% SDS-PAGE gels and transferred onto PVDF membranes. Membranes were blocked with 5% nonfat dry milk and incubated overnight at 4 °C with the following primary antibodies: rabbit polyclonal anti-villin-1 (carboxy terminus of human villin-1; 1:1000; catalog no. 2369, Cell Signaling, Boston, MA, USA); rabbit monoclonal anti-myosin light chain 2 (residues near the carboxy terminus of human myosin light chain 2; 1:1000; catalog no. 8505, Cell Signaling, Boston, MA, USA); mouse monoclonal anti-phospho-myosin light chain 2 (residues surrounding Ser19 of human myosin light chain 2; 1:1000; catalog no. 3675, Cell Signaling, Boston, MA, USA); and rabbit monoclonal anti-β-Actin (residues near the amino terminus of human β-actin; 1:1000; catalog no. 4970, Cell Signaling, Boston, MA, USA). After extensive washing with TBS-Tween, PVDF membranes were incubated for 2 h at room temperature with horseradish peroxidase-conjugated secondary antibody and developed an enhanced chemiluminescence (ECL) system. Immunoreactive proteins were visualized using ECL system (BIO-RAD, Hercules, CA, USA) in ChemiDoc XRS+ image acquisition system (BIO-RAD, Hercules, CA, USA), and densitometric quantification of detected bands was performed with Image Lab Software, version 6.1 (BIO-RAD, Hercules, CA, USA).

IAP activity was determined using the quantitative Alkaline Phosphatase Assay Kit (ab83369, Abcam, Cambridge, UK) with a measurement range of 10–250 µU and sensitivity of >10 µU. Villin expression was further assessed by immunohistochemistry using a mouse monoclonal anti-villin primary antibody (#ab201989, Abcam, Cambridge, UK). Sections that showed epithelial crypts and all layers of intestinal tissue were considered for analysis.

### 2.6. Fecal Microbiota Composition (FMC) Analysis

The collection, storage, and transportation of fecal material were performed according to standard procedures. Participants received prior instructions to ensure proper collection and avoid contamination given. The genomic DNA from fecal samples was extracted using the MoBio Power Soil DNA Isolation kit (Carlsbad, CA, USA). The 254-base-pair region corresponding to the V4 loop of the 16S rRNA gene was amplified for each isolated DNA sample using PCR primers F515 and R806 [21]. Sequencing was conducted on an Illumina MiSeq System with the 500-cycle MiSeq reagent kit V2 with paired-end 250 bp reads (Illumina Inc., San Diego, CA, USA).

Paired-end sequence reads were demultiplexed with MiSeq software 2.0 and were processed through quality control and analysis procedures following the MiSeq SOP using Mothur software (http://www.mothur.org, version 1.37.0, accessed on 23 November 2022) [22]. The number of paired-end sequences obtained for each sample ranged from 2,293,656 to 119,412. Briefly, paired reads were joined, sequencing errors were corrected, sequences were aligned against a reference database, chimeric sequences were removed, and the remaining sequences were taxonomically classified. Unique sequences were clustered into operational taxonomic units (OTUs) at a 97% sequence identity threshold. All sequence reads have been submitted to the SRA database under accession number SRP151928.

### 2.7. Statistical Analysis

The Shapiro–Wilk test was used to assess data normality. Results were expressed as the mean ± standard deviation or median [1st–3rd quartiles]. Group comparisons were performed using one-way ANOVA or Kruskal–Wallis tests, followed by Tukey’s and Dunn’s post hoc tests when significant F-ratios were observed. Pearson or Spearman correlation analyses were used to assess associations between intestinal permeability, histomorphometry, enzymatic activity, and protein expression in the intestinal epithelium and metabolic, inflammatory, and anthropometric variables.

For FMC analysis, Mothur was used to generate an OTU table and taxonomy file containing taxonomic information for each OTU. These data were used to plot the relative abundances of different taxonomic ranks. Differences in taxonomic composition between groups at the phylum and genus levels were assessed using ANOVA with a significance level of α = 0.05. The OTU table and taxonomy file were imported into R (version 3.6.1), and the phyloseq package [23] was used for alpha and beta diversity analyses. Rarefaction and MaAsLin 2 (Microbiome Multivariable Associations with Linear Models) analyses were performed by uploading a BIOM-formatted file and its respective metadata into the web platform MicrobiomeAnalyst (https://microbiomeanalyst.ca, accessed on 13 June 2025) [24]. For alpha and beta diversity analysis in phyloseq, sequencing read counts in each sample were normalized using the median sequencing depth. Pairwise comparisons of alpha-diversity metrics were performed using the Wilcoxon rank-sum test with Holm correction for *p*-value adjustment (α = 0.05) [25]. Beta-diversity analysis was conducted using Bray–Curtis dissimilarity and visualized with a principal coordinate analysis (PCoA) plot. Group differences in beta-diversity were evaluated using Permanova via the adonis function in vegan (phyloseq) with 999 permutations. For rarefaction analysis, sequences from each sample were rarefied to the lowest library size (30,776 sequences) and scaled using total sum scaling. Rarefaction plots and Good’s coverage metrics were generated for all samples. MaAsLin 2 analysis [26] was performed to determine pairwise differential abundances of OTUs among the NOB, DOB and CON groups. For this analysis, data from each sample were rarefied and scaled as described above and the Zero-Inflated Negative Binomial (ZINB) regression model [27] was used to calculate log2 fold changes in OTU abundances. Resulting *p*-values were controlled by False Discovery Rates (FDR) at 0.01. Statistical analyses were also performed using GraphPad Prism^®^ 5 software (GraphPad Software Inc., San Diego, CA, USA). The statistical significance level was set at *p* < 0.05.

## 3. Results

### 3.1. Clinical and Metabolic–Inflammatory Characteristics of the Participants

Regarding demographic characteristics and body composition (Table 1), the groups were similar in lean mass and waist-to-hip ratio (*p* > 0.06). The DOB group was older than the NOB group (*p* < 0.05). The proportion of females and the prevalence of hypertension was higher in DOB compared with CON (*p* < 0.02). In addition, both NOB and DOB showed higher body weight, BMI, waist and hip circumferences, and %fat mass than CON (*p* < 0.02). No significant differences were observed for total cholesterol and its fractions, triglycerides, AST, LPS, and LBP (*p* > 0.22). The DOB group had higher fasting PG than both CON and NOB (*p* < 0.05) and higher insulin, HOMA-IR, HbA1c, and ALT compared with CON (*p* < 0.05).

### 3.2. Assessment of Intestinal Epithelial Permeability: Histomorphometry, Enzymatic Activity, and Protein Expression

Histomorphometric parameters (*p* > 0.20) and protein expression (*p* > 0.16) in the intestinal epithelium were similar among the groups (Table 2). Notably, IAP activity was lower in DOB compared with CON (*p =* 0.02).

### 3.3. Fecal Microbiota Composition and Diversity Across Study Groups

**Figure 1:**
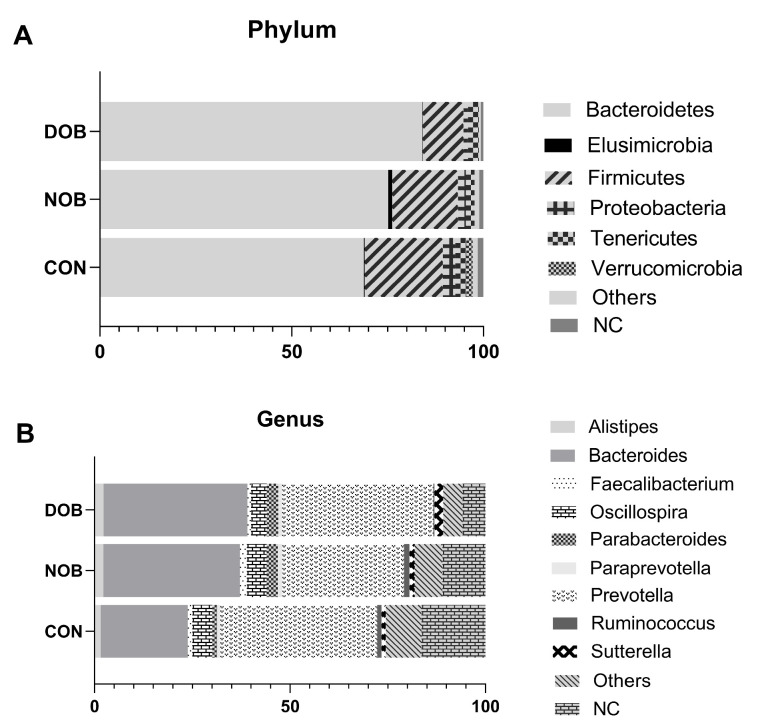
Taxonomic classification of operational taxonomic units (OTUs) obtained for the fecal microbiota of the study groups. Values are expressed as relative proportions, and those taxa that were present at less than 1% were grouped as others. Non-classified (NC) are OTUs that could only be classified as Bacteria. (**A**): OTUs classified at the Phylum level. (**B**): OTUs classified at the genus level.

Rarefaction analysis revealed that all samples achieved at least 99% coverage of the sampled microbial diversity (Supplementary Table S1), as also confirmed by the plateau observed in plots of species richness against sequence sample size (Supplementary Figure S1). Regarding FMC, there was considerable interindividual variability at both the phylum and genus taxonomic ranks (Supplementary Figures S2 and S3, respectively). The predominance phyla were Firmicutes and Bacteroidetes. When plotting the taxonomic composition at the phylum level for each group, lower relative abundances of Firmicutes, accompanied by a concomitant increase in Bacteroidetes, were observed in both NOB and DOB groups (Figure 1A). Additionally, the DOB group showed the lowest proportion of Firmicutes; however, these differences were not statistically significant (Figure 1A).

A substantial proportion of OTUs (ranging from 1 to 38%) could not be assigned to the genus level, and the most abundant genera were *Bacteroides* and *Prevotella* (Supplementary Figure S3). When taxonomic composition was analyzed at the group level, *Bacteroides* and *Prevotella* remained the most prevalent genera, followed by *Oscillospira* (Figure 1B). Across groups, the proportion of OTUs unclassified at the genus level ranged from 6 to 17% (Figure 1B). No statistically significant differences in genus abundance were detected between groups.

Different alpha-diversity metrics were calculated for the groups (Supplementary Figure S4), and no statistically significant changes in diversity were observed among them. Differences in beta-diversity were assessed by calculating Bray–Curtis dissimilarity distances, which were used for multivariate analysis and visualized using a PCoA plot (Supplementary Figure S5). No statistically significant differences in FMC in beta-diversity analysis were observed among groups. At higher taxonomic levels, differences in FMC were not detected among groups, and no significant differences were observed for either alpha- or beta-diversity comparisons. Therefore, we proceeded to investigate more subtle changes at the OTU level.

### 3.4. OTU Differential Abundance Profiles Assessed with MaAsLin 2

The differential abundance of OTUs among groups was analyzed using MaAsLin 2 with the ZINB model. Pairwise comparisons were performed between groups. The comparison between NOB and DOB did not reveal any differentially abundant OTUs. However, when comparing NOB with CON, one OTU (OTU104), assigned to the genus *Bacteroides* (Table 3), showed an increased abundance in the NOB group. In contrast, when comparing the DOB group with CON, eight OTUs, all assigned to the phylum Firmicutes, were underrepresented in the DOB group (Table 3). The least abundant OTU in the DOB group is OTU183, identified as *Clostridium celatum*. The second least abundant, OTU028 could only be assigned to the phylum level. OTU125 was assigned to the family Ruminococcaceae, OTU278 was assigned to the genus *Veillonella*, OTU261 and OTU168 could be identified as *Blautia obeum* and *Ruminococcus gnavus*, respectively, and OTUs 243 and 272 were assigned to the genus *Coprococcus*. Notably, except for OTU028, all other OTUs were affiliated with the order Clostridiales.

### 3.5. Associations Between Inflammatory and Intestinal Permeability Markers and Metabolic–Anthropometric Parameters

Table 4 presents the correlations between inflammatory markers, intestinal permeability, histomorphometric parameters, enzymatic activity, and protein expression in intestinal epithelium with metabolic and anthropometric markers. Notably, LPS and LBP levels were positively correlated with body weight (r = 0.32; *p* < 0.05) and waist-to-hip ratio (r = 0.37; *p* < 0.05), respectively. The villus-to-crypt relation was negatively associated with BMI (r = −0.44; *p* < 0.01). IAP activity was negatively associated with several biochemical markers, including fasting PG and HbA1c (r = −0.50 and r = −0.62; *p* < 0.01, respectively). Finally, β-actin expression was inversely correlated with BMI (r = −0.32; *p* < 0.05), fasting PG (r = −0.49; *p* < 0.01), insulin (r = −0.49; *p* < 0.01), and HOMA-IR (r = −0.55; *p* < 0.001).

## 4. Discussion

This cross-sectional study, which involved 46 participants divided into three groups, should be regarded as a pilot investigation. Nevertheless, several key findings were identified, as follows: (a) the proportions of Bacteroidetes and Firmicutes—the most prevalent phyla in FMC—as well as alpha- and beta-diversity, did not differ among groups; (b) differences in OTU abundance were observed between the NOB and DOB groups compared with CON; (c) histomorphometric parameters and the expression of junctional proteins in the duodenal epithelium did not differ among groups; (d) no significant differences in LPS and LBP levels were detected among groups; (e) IAP activity was significantly lower in the DOB group compared with CON; (f) IAP was negatively correlated with plasma glucose and HbA1c; and (g) expression of β-actin from intestinal epithelium was inversely correlated with plasma glucose, insulin, HOMA-IR and BMI. As anticipated, the DOB group displayed the poorest metabolic profile, characterized by elevated fasting glucose, insulin, HbA1c, and HOMA-IR, indicating significant metabolic dysfunction and insulin resistance.

FMC analysis revealed considerable interindividual variability and no significant differences among the groups in the most common phyla, Bacteroidetes and Firmicutes. While some studies have reported associations between adiposity and FMC, particularly regarding these phyla [28], others have failed to establish this link [29,30]. Similarly, population-based studies exploring the relationship between gut microbiota and T2D have yielded conflicting results [31]. High interindividual variability in FMC may result from dietary, environmental, and immunological factors, as well as variations in gastric pH and intestinal motility [32], factors that this study could not entirely account for.

At the genus level, interindividual variability remained high, with Bacteroides, Prevotella, and Oscillospira being the most abundant genera. Genetic influences, age, environment, diet and lifestyle habits may contribute to these variations and play significant roles in metabolic disorders and their treatment [33,34]. While FMC remains an essential aspect requiring further and deeper investigation, the challenges posed by these confounding factors underscore the need for standardized and accurate analytical methods.

The Brazilian population exhibits pronounced heterogeneity in the proportions of European, African, and Indigenous ancestry across different geographic regions, which may influence the distribution of genetic variants associated with chronic diseases and affect both disease pathophysiology and therapeutic responses [35]. Among the human colonic microbiota, commensal bacteria from the families *Lachnospiraceae* and *Ruminococcaceae*, within the *Clostridiales* order, are major producers of short-chain fatty acids (SCFAs), particularly butyrate. These metabolites are critical for maintaining intestinal epithelial integrity, modulating host inflammatory pathways, and improving insulin sensitivity [36,37]. Multiple studies have demonstrated that individuals with T2D exhibit reduced microbial diversity within the *Clostridiales*, especially a marked depletion of strains such as *Faecalibacterium prausnitzii,* which are involved in anti-inflammatory effects [38,39]. This dysbiotic shift contributes to a pro-inflammatory gut milieu and heightened insulin resistance, thereby exacerbating disease progression [40]. Experimental models further support these findings, showing that reintroduction of specific *Clostridiales* strains ameliorates glucose intolerance and reduces systemic inflammation [40]. Despite the extensive genetic admixture within the Brazilian population, our findings corroborate previous evidence, demonstrating that patients with dysglycemia consistently exhibit a reduced abundance of *Clostridiales*, underscoring the conserved relevance of this microbial group in the disease’s pathophysiology across diverse genetic backgrounds.

The gut microbiota plays a pivotal role in maintaining intestinal barrier integrity. Microbial dysbiosis, characterized by alterations in composition and diversity, can lead to intestinal barrier dysfunction [41,42]. This dysfunction results from disrupted expression of tight junction proteins or impaired intra- and extracellular molecular interactions, allowing bacterial antigens and microorganisms to translocate from the gut lumen into circulation [43,44]. Part of the systemic low-grade inflammation observed in obesity and T2D has been linked to intestinal barrier disruption, primarily through increased intestinal permeability, which is often reflected in elevated LPS levels [45,46]. Circulating LPS binds to LBP, activating CD14 receptors [47] and forming a complex that interacts with Toll-like receptor-4 on macrophages and adipose tissue, triggering proinflammatory gene transcription [47,48]. Elevated LPS levels in individuals with obesity and T2D contribute to metabolic endotoxemia [9], which, in turn, downregulates intestinal tight junction proteins such as occludin and zonula occludens-1 [9], further compromising the epithelial barrier. Additionally, biomarkers of intestinal permeability, including LBP and zonulin, have been found at higher levels in individuals with obesity compared with controls [49]. In our study, LPS levels were positively correlated with body weight, and LBP levels correlated with the waist-to-hip ratio. However, we did not observe significant differences in circulating LPS and LBP levels among groups. This could be attributed to the lack of significant differences in FMC (in terms of genera, phyla, and alpha- and beta-diversities), as well as to the sample size, unmeasured confounding factors, and methodological aspects. Notably, many previous studies have assessed LPS and LBP levels [14,49] in the context of a postprandial inflammation, typically induced by lipid-rich meals [41], which may amplify these markers and accentuate group differences. In contrast, our study assessed LPS and LBP levels in the fasting state.

IAP is a duodenal brush-border enzyme that detoxifies LPS by dephosphorylating its lipid A region [50], thereby mitigating LPS-induced inflammation [51]. While high-fat diets have been shown to increase IAP activity in rats, it remains unclear whether decreased IAP activity is a cause or consequence of inflammatory stimuli [14]. Given its inverse relationship with intestinal permeability, IAP may play a role in preventing metabolic syndrome [52]. In our study, IAP activity was inversely correlated with fasting glucose and HbA1c levels, and was significantly reduced in the DOB group compared with CON. This reduction in IAP activity may have impaired LPS clearance, exacerbating the dysglycemic inflammatory state. Notably, the finding aligns with previous observations in animal models [14], suggesting a potential role of IAP in metabolic disease. Indeed, previous studies have demonstrated that patients with T2D exhibit reduced levels of IAP in stool samples compared with healthy individuals [53]. Conversely, IAP deficiency has been associated with an increased risk of T2D, whereas oral IAP supplementation prevented the development of T2D [54].

Histomorphometric analysis revealed an inverse correlation between the villus-to-crypt ratio and BMI. Among the duodenal proteins analyzed, β-actin emerged as a key player, showing inverse correlations with BMI and metabolic markers. These findings support the hypothesis that intestinal barrier disruption contributes to the pathogenesis of obesity and dysglycemia. However, due to cross-sectional design of the study, it is not possible to establish a causal relationship between the reduced expression of β-actin in the intestinal epithelium and the worsening of the metabolic dysfunction. Therefore, further investigation is required to clarify the potential causal link between duodenal epithelial β-actin expression and metabolic disorders. β-actin, a cytoplasmic actin isoform, may exert a protective effect by maintaining intestinal epithelial barrier integrity through stabilizing tight junctions and adherens junctions via its anchorage to junctional complexes [55]. Higher levels of β-actin were observed in individuals with lower BMI, lower insulin resistance, and reduced inflammation.

Some limitations of our study need to be mentioned. The first concerns its cross-sectional design, which limits inferences regarding cause-and-effect relationships. Although certain inferences can be drawn, the findings should be interpreted with caution and not generalized. We highlight that further prospective studies involving a larger number of participants are needed to investigate these associations. The groups were not fully age-matched, which may have influenced the results, as aging is a recognized risk factor for metabolic dysfunction through multiple mechanisms. Given the numerous contradictions in literature, we aimed to demonstrate, for the first time in humans—particularly within our genetically admixed population—associations between intestinal parameters and metabolic dysfunction. These findings may generate new hypotheses to be explored in future prospective and longitudinal studies. Another minor limitation should be acknowledged: the predominance of female participants, which reflects the demographic profile of the clinical population from which the sample was recruited. Although this sex imbalance may limit the precision of sex-based comparisons, it accurately represents the real-world epidemiology of obesity and healthcare-seeking behavior in outpatient settings. Furthermore, functional validation of histomorphometric and protein expression analyses was not performed. Additional mechanistic follow-up regarding group difference in IAP activity could have provided further insights. Ideally, a more detailed characterization of the patients’ dietary intake (such as nutrient content or dietary quality) and other lifestyle-related factors, as well as genetic influences, age, sex hormones, and environmental factors not controlled for in our study, would have offered valuable information.

## 5. Conclusions

We demonstrated that IAP activity is lower in individuals with obesity and dysglycemia, reinforcing its potential role in disease pathogenesis. Additionally, IAP activity was inversely associated with insulin resistance markers, further linking it to metabolic syndrome. β-actin also emerged as a significant factor in intestinal barrier homeostasis, given its association with metabolic variables. These findings occurred alongside subtle alterations in FMC, particularly within the *Clostridiales* order. Given the challenges inherent to FMC research, we emphasize the need for standardized methodologies in future studies. Our findings provide a foundation for future clinical trials investigating IAP and β-actin as potential therapeutic targets in metabolic disease. Longitudinal studies are warranted to elucidate the causal relationships between IAP activity, intestinal barrier function, and metabolic outcomes. Furthermore, interventional trials should evaluate the therapeutic potential of IAP supplementation or modulation.

## Figures and Tables

**Table 1:** Demographic characteristics, body composition, and metabolic and inflammatory profile of the participants.

Variables	Pooled Sample (n = 46)	CON (n = 16)	NOB (n = 15)	DOB (n = 15)	*p*-Value
Demographic characteristics					
Age (years)	38.3 ± 7.8	39.2 ± 7.4	33.0 ± 7.8	41.7 ± 6.2 †	0.02
Females (n, %)	38 (82.6)	10 (62.5)	13 (86.6)	15 (100) *	0.02
Weight (kg)	89.4 ± 16.1	71.3 ± 11.2	98.9 ± 12.8 *	93.2 ± 12.0 *	<0.001
BMI (kg/m^2^)	32.6 ± 5.1	25.5 ± 2.3	34.9 ± 3.4 *	35.2 ± 2.6 *	<0.001
Waist circumference (cm)	104.6 ± 11.4	89.5 ± 7.4	112.1 ± 6.1 *	108.4 ± 7.0 *	<0.001
Hip circumference (cm)	115 [110–122]	99 [95.7–107]	116 [113–127] *	114.5 [110–122] *	0.02
Waist-to-hip ratio	0.9 [0.87–0.95]	0.88 [0.82–0.91]	0.94 [0.88–0.95]	0.90 [0.88–0.99]	0.15
Hypertension (n, %)	10 (21.7)	0 (0)	2 (13.3)	8 (53.3) *	<0.001
Body composition					
Lean mass (kg)	53.3 [47.38–58.3]	48.1 [43.0–56.6]	56.2 [49.8–61.5]	53 [50.9–58.6]	0.06
Fat mass (%)	38.5 [28.9–41.3]	25.6 [23.7–28.5]	41.3 [36.6–45.0] *	40.7 [38.1–41.3] *	<0.001
Metabolic/inflammatory profile					
Fasting glucose (mg/dL)	104 [96.5–115.5]	99 [93–109]	101 [94.7–104.5]	118.5 [113.8–152.5] *†	<0.001
Insulin (pg/mL)	299 [158.5–453]	136 [89.8–276]	310.5 [228.8–390]	451 [319–557] *	0.001
HOMA-IR	1.79 [0.93–3.05]	0.79 [0.51–1.77]	1.78 [1.43–2.46]	3.21 [1.78–6.03] *	0.001
HbA1c (%)	5.4 [5.3–5.9]	5.3 [5.0–5.4]	5.5 [5.3–5.6]	6.1 [6.0–8.4] *	<0.001
Total cholesterol (mg/dL)	179.2 ± 31.87	183.4 ± 33.9	178.7 ± 38.8	175 ± 23.4	0.34
HDL-cholesterol (mg/dL)	49 [39.50–57.75]	51.5 [42–59.7]	54 [40–62.5]	46 [39–49]	0.22
LDL-cholesterol (mg/dL)	108 [93–122.5]	106 [93–133]	104 [88–117.5]	111 [93–119]	0.85
Triglycerides (mg/dL)	98 [71.8–43.8]	96.5 [74.2–131.5]	83 [65–135.5]	118 [86–155]	0.22
AST (U/mL)	17 [13–22]	16.5 [12–19]	16.5 [13.5–22]	18.5 [13–25]	0.64
ALT (U/mL)	17.5 [11.8–26]	11 [9–23.5]	17 [12–24]	24.5 [16.2–34.7] *	0.05
LPS (U/mL)	1.10 [0.55–1.45]	1.02 [0.47–1.47]	1.17 [0.91–1.50]	1.10 [0.53–1.65]	0.60
LBP (μg/mL)	23.28 [20.79–27.72]	23.5 [19.4–26.6]	23.5 [22.0–30.6]	22.9 [20.8–27.6]	0.26

Abbreviations: CON, Control group; NOB, Normoglycemic with obesity group; DOB, Dysglycemic and obesity group; BMI, Body mass index; HOMA-IR, Homeostasis model assessment for insulin resistance; HbA1c, Glycated Hemoglobin type A1c; LDL-c, Low-density lipoprotein cholesterol; HDL-c, High-density lipoprotein cholesterol; ALT, Alanine aminotransferase; AST, Aspartate aminotransferase; LPS, Bacterial lipopolysaccharide; LBP, LPS-binding protein. *p*-values of One-way ANOVA, Kruskal–Wallis, or chi-square test; results expressed as mean ± standard deviation, medians [1st–3rd quartiles] or n (%). *: significant difference vs. CON (*p* < 0.05). †: significant difference between NOB vs. DOB (*p* < 0.05).

**Table 2:** Markers of intestinal epithelial permeability (histomorphometry, enzymatic activity, and protein expression in duodenal biopsies).

Variables	Pooled Sample (n = 44)	CON (n = 14)	NOB (n = 15)	DOB (n = 15)	*p*-Value
Histomorphometric analysis					
Total epithelial thickness (µm)	13.76 [11.18–17.20]	14.45 [12.21–17.20]	13.07 [11.18–17.20]	13.76 [11.18–15.48]	0.53
Intestinal villus height (µm)	12.04 [9.46–14.62]	12.04 [10.75–15.90]	12.04 [9.46–14.62]	12.04 [9.29–13.76]	0.53
Villus diameter I (µm)	3.96 [3.44–4.30]	3.78 [3.44–4.73]	3.78 [3.44–4.30]	4.13 [3.44–5.16]	0.51
Villus diameter II (µm)	1.03 [1.03–1.38]	1.03 [1.03–1.20]	1.03 [1.03–1.38]	1.20 [1.03–1.72]	0.25
Crypt height (µm)	1.38 [0.86–1.72]	1.03 [0.86–1.72]	1.38 [0.86–1.89]	1.55 [1.03–1.72]	0.78
Villus-to-crypt ratio (µm)	9.47 ± 3.62	10.80 ± 2.83	9.38 ± 4.54	8.33 ± 3.12	0.20
Enzymatic activity					
Intestinal Alkaline phosphatase (U/mL)	0.58 ± 0.29	0.75 ± 0.23	0.56 ± 0.27	0.43 ± 0.31 *	0.02
Protein expression					
Villin-1	0.82 [0.74–0.86]	0.83 [0.75–0.89]	0.81 [0.74–0.86]	0.81 [0.74 0.86]	0.72
Myosin-2 light chain	0.19 [0.08–0.43]	0.25 [0.05–0.45]	0.19 [0.09–0.38]	0.15 [0.08–0.33]	0.86
Phosphomyosine	0.15 [0.04–0.46]	0.18 [0.03–0.62]	0.25 [0.04–0.51]	0.09 [0.05–0.25]	0.56
β-actin	0.11 [0.07–0.23]	0.20 [0.08–0.43]	0.11 [0.06–0.30]	0.10 [0.04–0.15]	0.16

Abbreviations: CON, Control group; NOB, Normoglycemic with obesity group; DOB, Dysglycemic and obesity group. *p*-values of One-way ANOVA or Kruskal–Wallis test; results expressed as mean ± standard deviation or medians. [1st–3rd quartiles]; *: significant difference vs. CON (*p* < 0.05).

**Table 3:** MaAsLin 2 results.

		NOB vs. CON				
Feature	Log2FC	St.Error	*p*-Value	FDR	Taxonomy	
OTU104	2.73	0.687	7.07 × 10^−5^	0.0019	p_Bacteroidetes; c_Bacteroidia; o_Bacteroidales; f_Bacteroidaceae; g_*Bacteroides*	
		DOB vs. CON				
Feature	Log2FC	St.Error	*p*-value	FDR	Taxonomy	
OTU028x	−3.91	0.707	3.13 × 10^−8^	7.58 × 10^−6^	p_Firmicutes	
OTU243	−2.33	0.483	1.40 × 10^−6^	0.000127	p_Firmicutes; c_Clostridia; o_Clostridiales; f_Lachnospiraceae; g_*Coprococcus*	
OTU272	−2.19	0.456	1.58 × 10^−6^	0.000127	p_Firmicutes; c_Clostridia; o_Clostridiales; f_Lachnospiraceae; g_*Coprococcus*	
OTU278x	−3.59	0.761	2.40 × 10^−6^	0.000145	p_Firmicutes; c_Clostridia; o_Clostridiales; f_Veillonellaceae; g_*Veillonella*	
OTU183x	−4.49	0.999	6.92 × 10^−6^	0.000335	p_Firmicutes; c_Clostridia; o_Clostridiales; f_Clostridiaceae; s_*Clostridium celatum*	
OTU168x	−2.21	0.497	8.97 × 10^−6^	0.000362	p_Firmicutes; c_Clostridia; o_Clostridiales; f_Lachnospiraceae; s_*Ruminococcus gnavus*	
OTU125x	−3.66	0.901	4.85 × 10^−5^	0.00153	p_Firmicutes; c_Clostridia; o_Clostridiales; f_Ruminococcaceae	
OTU261x	−2.36	0.583	5.07 × 10^−5^	0.00153	p_Firmicutes; c_Clostridia; o_Clostridiales; f_Lachnospiraceae; s_*Blautia obeum*	

Abbreviations: CON, Control group; NOB, Normoglycemic with obesity group; DOB, Dysglycemic and obesity group.

**Table 4:** Associations between inflammatory and intestinal epithelial permeability markers and metabolic and anthropometric markers.

	Metabolic Markers				Anthropometric Markers		
	Fasting Glucose	Insulin	HOMA-IR	HbA1c	Body Weight	BMI	Waist/Hip Ratio
Intestinal permeability							
LPS (EU/mL)	−0.01	−0.10	−0.09	0.07	0.32 *	0.27	−0.05
LBP (μg/mL)	−0.03	0.08	0.10	0.26	0.03	0.05	0.37 *
Histomorphometric analysis							
Total epithelial thickness (µm)	0.03	−0.03	−0.10	0.05	0.05	−0.07	0.29
Intestinal villus height (µm)	0.04	−0.01	−0.07	−0.05	−0.03	−0.18	0.29
Villus diameter I (µm)	0.20	0.11	0.17	0.03	0.01	0.03	−0.22
Villus diameter II (µm)	−0.03	0.10	0.06	0.32	0.13	0.26	−0.12
Crypt height (µm)	0.30	0.21	0.20	0.31	0.19	0.27	0.16
Villus-to-crypt ratio (µm)	−0.23	−0.29	−0.31	−0.33	−0.27	−0.44 †	−0.03
Enzymatic activity							
IAP (U/mL)	−0.50 †	−0.23	−0.30	−0.62 †	−0.09	−0.23	−0.33
Protein expression							
Villin-1	0.17	0.01	0.06	0.05	−0.01	0.01	−0.28
Myosin-2 light chain	−0.22	−0.01	−0.03	−0.24	−0.09	−0.14	0.26
Phosphomyosine	−0.14	−0.01	−0.11	−0.01	0.15	0.09	−0.04
β-actin	−0.49 †	−0.49 †	−0.55 Φ	−0.24	−0.26	−0.32 *	0.03

Abbreviations: HOMA-IR, Homeostasis model assessment of insulin resistance; HbA1c, Glycated Hemoglobin type A1c; BMI, Body mass index; LPS, Bacterial lipopolysaccharide; LBP, LPS-binding protein; IAP, Intestinal alkaline phosphatase. *: *p* < 0.05 †: *p* < 0.01; Φ: *p* < 0.001.

## Data Availability

The datasets used to support the findings of this study are available from the corresponding author upon request.

## Supplementary Materials

The following supporting information can be downloaded at: https://www.mdpi.com/article/10.3390/nu17213380/s1. Figure S1. Rarefaction curves of individual patients from the groups. Sequences from each sample were rarefied to the lowest library size (30,776 sequences) and scaled using total sum scaling and species richness was plotted against sequence sample size. Figure S2. Taxonomic classification of operational taxonomic units (OTUs) obtained for the fecal microbiota of individual patients from the groups at the Phylum level. Values are expressed as relative proportion, and those taxa that were present at less than 1% and/or that could only be classified as Bacteria were grouped as others. Figure S3. Taxonomic classification of operational taxonomic units (OTUs) obtained for the fecal microbiota of individual patients from the groups at the Genus level. Values are expressed as a relative proportion, and those taxa that were present at less than 1% were grouped as others. Non-classified (NC) are OTUs that could only be classified as Bacteria. Figure S4. Alpha-diversity metrics for study groups. Operational taxonomic units (OTUs) counts were normalized as described in materials and methods and diversity was calculated with the R package phyloseq (v.1.52.0). Box-plots show the distribution of individual values and the median for each group. No statistically significant differences were observed between the groups for any of the metrics. Figure S5. Beta diversity PCoA plot of Bray–Curtis dissimilarity distances for the fecal microbiota of individual patients from study groups. Operational taxonomic units (OTUs) counts were normalized as described in material and methods and the distance between the individual samples was calculated with the R package phyloseq. The first axis of the PCoA explains 35.6% of the variance, while the second axis explains 13.3% of the variance. No statistically significant differences in beta-diversity were observed among groups. Table S1: Rarefaction Analysis.

## Abbreviations

The following abbreviations are used in this manuscript:

CVD	Cardiovascular diseases
T2D	Type 2 diabetes *mellitus*
LPS	Lipopolysaccharide
LBP	LPS-binding protein
FMC	Fecal microbiota composition
IAP	Intestinal alkaline phosphatase
OTUs	Operational taxonomic units
