# Supplementary figures and images for: Gut Microbiota, Intestinal Barrier Function, and Metabolism Across Adiposity and Glucose Tolerance
Source: Nutrients. 2025 Oct 28;17(21):3380. doi: 10.3390/nu17213380 (PMC12610977; doi:10.3390/nu17213380)

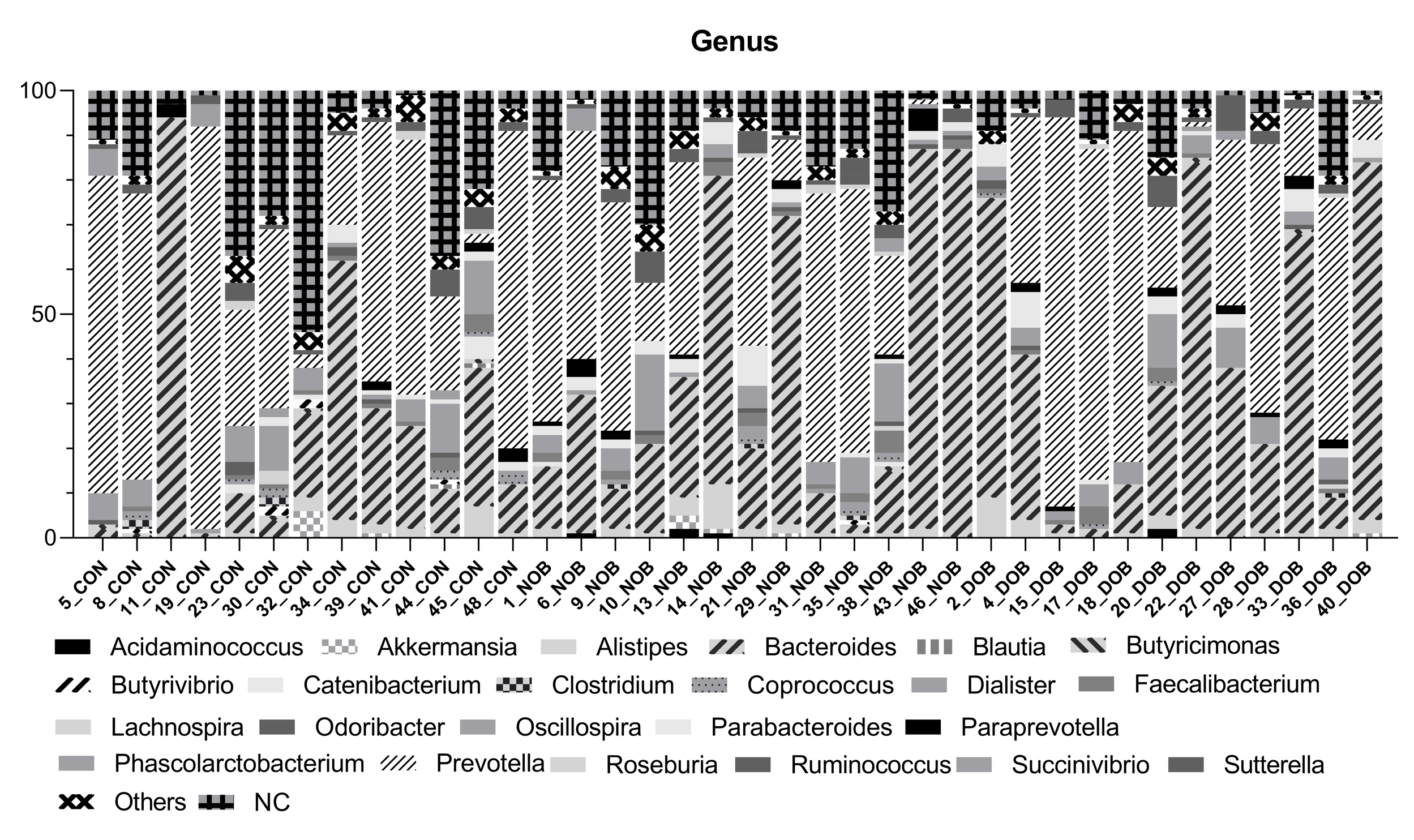

Supplement: Supplementary file 1 [file nutrients-17-03380-s001.zip › Supplementary FigureS3.jpg]

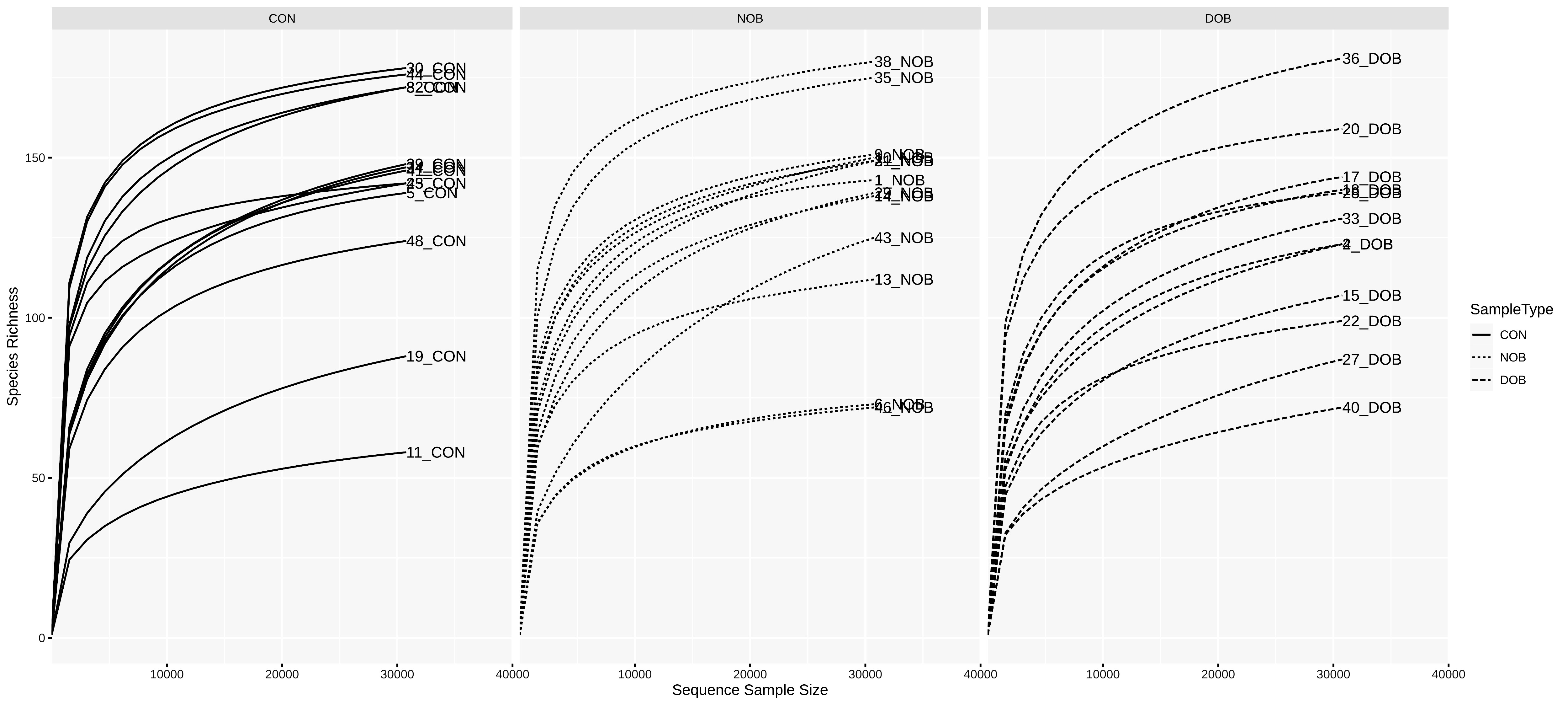

Supplement: Supplementary file 1 [file nutrients-17-03380-s001.zip › SupplementaryFigureS1.jpg]

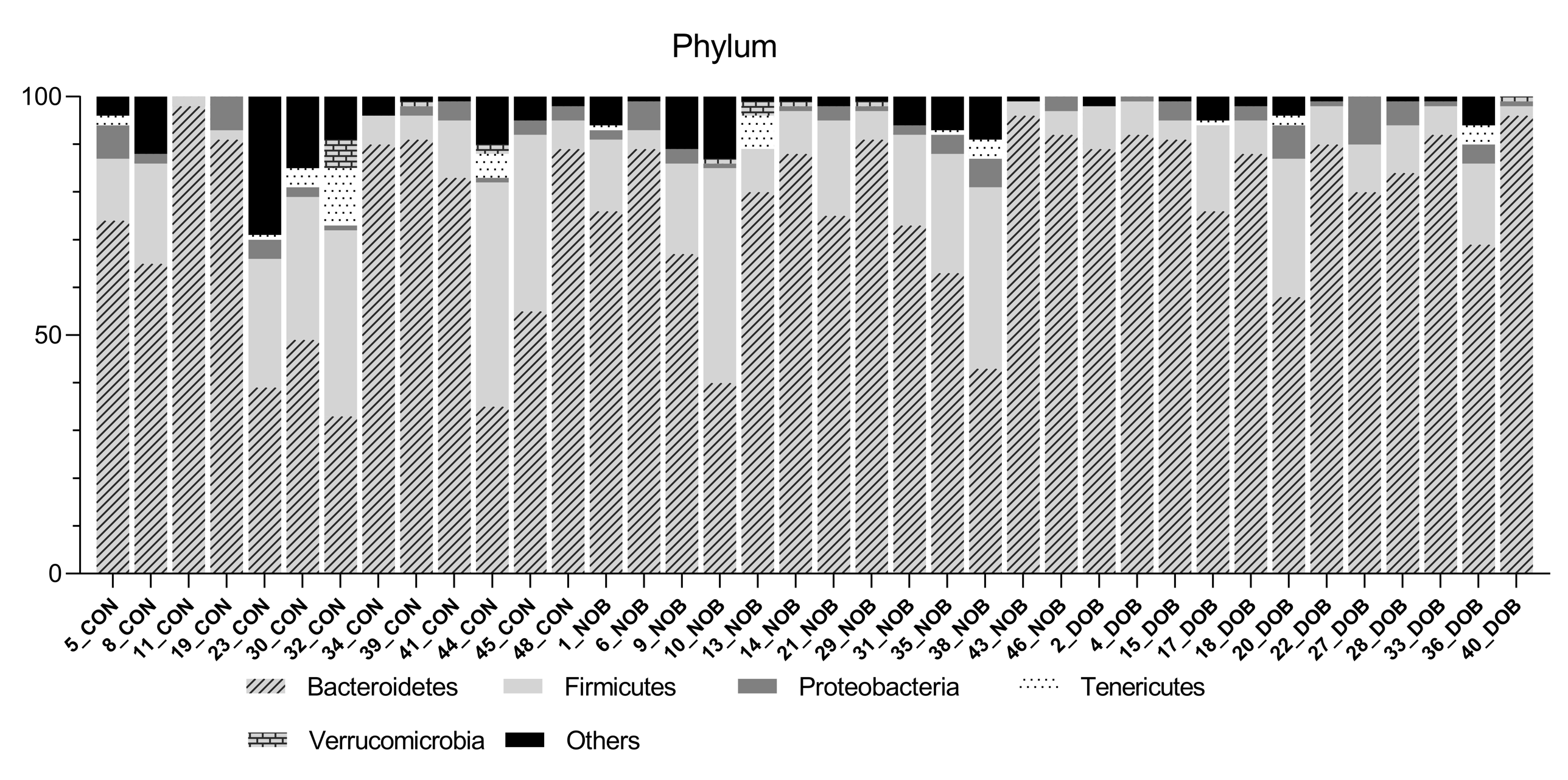

Supplement: Supplementary file 1 [file nutrients-17-03380-s001.zip › SupplementaryFigureS2.jpg]

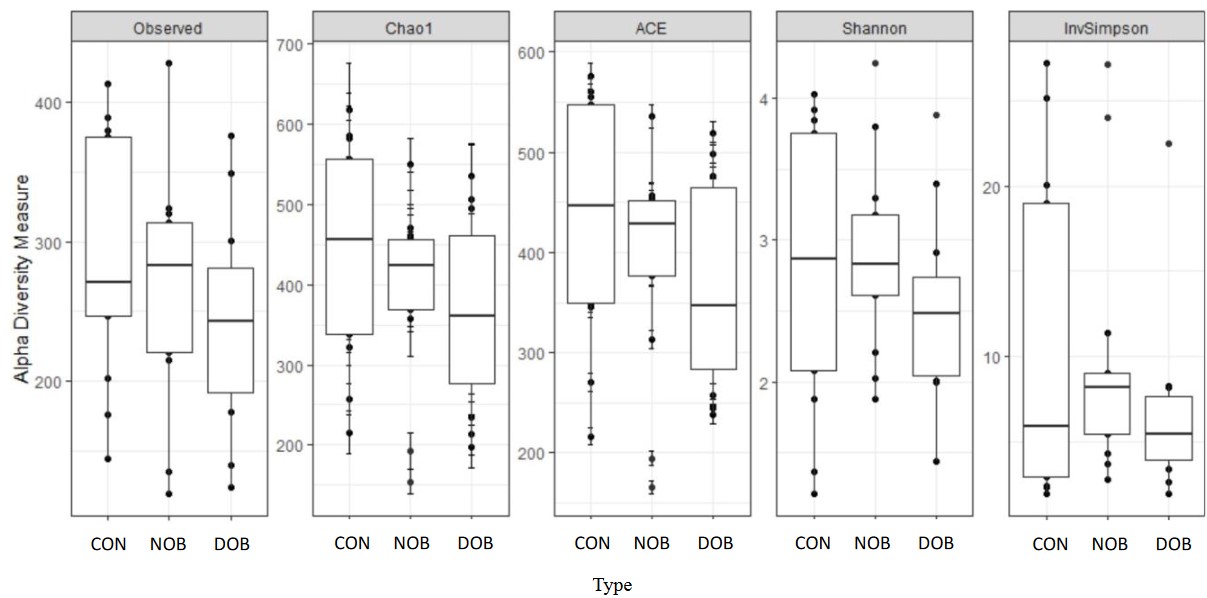

Supplement: Supplementary file 1 [file nutrients-17-03380-s001.zip › SupplementaryFigureS4.jpg]

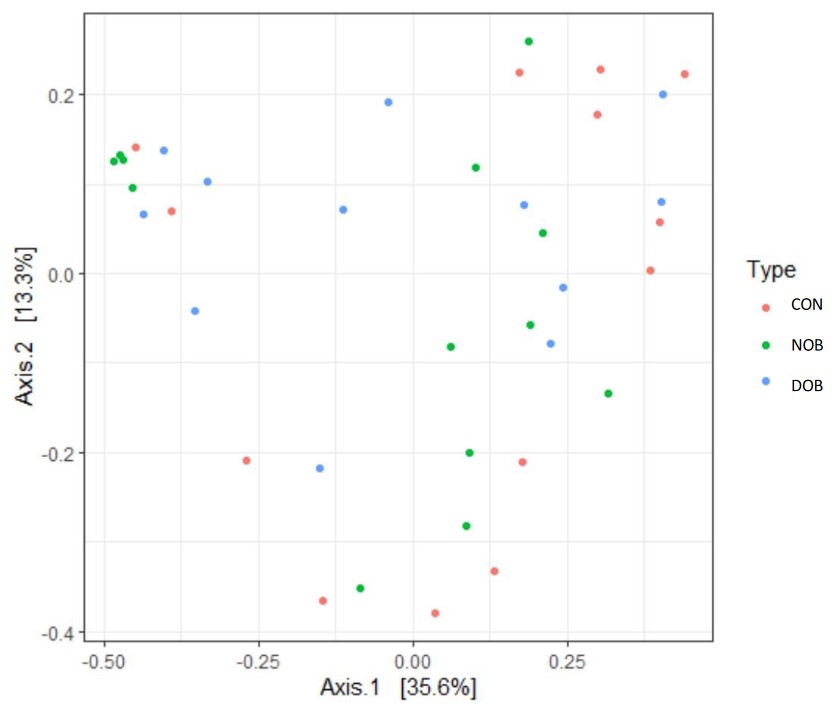

Supplement: Supplementary file 1 [file nutrients-17-03380-s001.zip › SupplementaryFigureS5.jpg]
